# Comprehensive Transcriptome Analysis of Patients With Keratoconus Highlights the Regulation of Immune Responses and Inflammatory Processes

**DOI:** 10.3389/fgene.2022.782709

**Published:** 2022-02-25

**Authors:** Xiao Sun, Hao Zhang, Mengyuan Shan, Yi Dong, Lin Zhang, Luxia Chen, Yan Wang

**Affiliations:** ^1^ School of Medicine, Nankai University, Tianjin, China; ^2^ Tianjin Medical University, Tianjin, China; ^3^ Tianjin Key Laboratory of Ophthalmology and Visual Science, Tianjin Eye Hospital, Tianjin, China

**Keywords:** keratoconus, transcriptome, cornea, peripheral blood, immune, inflammatory

## Abstract

Keratoconus (KTCN), characterized by the steeper curvature of the cornea and the thinner central corneal thickness, was a degenerative corneal ectasia with ambiguous etiology and mechanism. We aim to reveal underlying pathological mechanisms of KTCN by multi-level transcriptomic, integrative omics analyses. We performed RNA-sequencing on corneal epithelial samples from seven patients and seven control donors, as well as peripheral matched blood samples from three of the patients and three control donors. After RNA extraction, library construction, and sequencing, differentially expressed genes and splicing events were identified, followed by over-representation analysis. In total, 547 differential expressed genes were identified in KTCN corneal epithelial samples, which were mainly enriched in immune responses and inflammatory processes. WGCNA module analysis, the transcriptomic analysis of peripheral blood samples, multiple omics data, and the meta-analysis of GEO samples confirmed the involvement of immune and inflammatory factors. Besides, 190 and 1,163 aberrant splicing events were identified by rMATS combined with CASH methods in corneal epithelial and blood samples with KCTN. In conclusion, this comprehensive transcriptome analysis of KTCN patients based on patients’ tissue and blood samples uncovered a significant association between immune-inflammatory genes and pathways with KCTN, highlighting the contribution of the perturbed immune signaling to the pathogenesis of KCTN. Our study suggested the importance of measures to control inflammation in the treatment of KCTN.

## Introduction

Keratoconus (KTCN) is a corneal ectatic disorder characterized by a steeper curvature of the cornea and a thinner central corneal thickness. The global average prevalence of KTCN is 1.38/1,000, with great regional variations, ranging from 0.17/1,000 in Russia to 47.99/1,000 in Saudi Arabia (Hashemi et al., 2020). Current interventions for KTCN, such as contact lens use, corneal ring implantation, and collagen cross-linking, can only slow the progression of the disease. Currently, KTCN cannot be cured by any treatment method. Thus, it is important to understand the fundamental pathogenesis which may be helpful for the development of effective therapeutic interventions.

The exact cause of KTCN is not completely understood. Proteomics studies revealed decreased expression of structural proteins (Chaerkady et al., 2013) and increased expression of degradative enzymes. Pathway analyses indicated the involvement of genes related to extracellular matrix organization (Kabza et al., 2017), transforming growth factor β signaling, WNT pathway (You et al., 2018), and nuclear factor erythroid 2–related factor 2-regulated network (Shinde et al., 2020). In recent years, omics studies improved our understanding of the molecular mechanisms of KTCN (McKay et al., 2016; Kabza et al., 2019; Karolak et al., 2020).

Although KTCN was previously considered a non-inflammatory disease, there is increasing evidence supporting the involvement of inflammation and immune responses in KTCN. Various studies found inflammatory mediators in the corneal tissue and tears of patients with KTCN, including interleukin (IL)-1β (Sorkhabi et al., 2015), IL-6, tumor necrosis factor (TNF)-α (Lema et al., 2009), matrix metallopeptidase-9 (Balasubramanian et al., 2012a), and nuclear factor kappa B. Molecules involved in the immune response, such as lactoferrin (LTF), zinc-α2-glycoprotein, and immunoglobulin kappa chain, are downregulated in the tears of patients with KTCN. The serum expression levels of Toll-like receptors 2 and 4 in monocytes and neutrophils were significantly higher in patients with KTCN compared to control subjects (Sobrino et al., 2017). The latest literature shows that KTCN is positively correlated with a variety of immune-mediated diseases, such as Hashimoto’s thyroiditis and inflammatory skin conditions (Claessens et al., 2021).

Our study aimed to determine the underlying pathological mechanisms of KTCN by multi-level transcriptomic study and integrative omics analyses. To the best of our knowledge, this is the first comparative transcriptomic study of corneal epithelial and blood samples from patients with KTCN and control donors. Through bioinformatics analyses, real-time polymerase chain reaction (RT-PCR) validation, and co-analysis with published omics data and GEO data, we revealed the involvement of immune and inflammatory factors in KTCN pathogenesis. In addition, we analyzed alternative splicing events which may contribute to the pathophysiology of KTCN and made a prediction for potential drugs associated with KTCN.

## Materials and Methods

### Samples

This study conformed to the Declaration of Helsinki and was approved by the Research Ethics Committee of Tianjin Eye Hospital (KY202107). All participants provided written informed consent. Patients who were diagnosed with KTCN and underwent de-epithelized collagen cross-linking at Tianjin Eye Hospital from May 2020 to May 2021 were enrolled. Controls were sex-matched patients undergoing laser-assisted subepithelial keratomileusis surgery for mild myopia without ocular and other systemic diseases. All participants underwent a complete medical history assessment, general examination, and specialist examination. A total of seven KTCN corneal epithelial tissues, three KTCN blood samples, seven control corneal epithelial tissues, and three control blood samples were collected. The removed epithelium was immediately submerged in RNALater (Qiagen, Germany) or immediately subjected to the workflow of RNA extraction. Peripheral blood was collected in ethylenediaminetetraacetic acid-containing tubes, and peripheral blood mononuclear cells (PBMCs) were isolated using red blood cell lysis buffer (TianGen, Beijing).

### RNA Isolation, cDNA Library Construction, and Sequencing

RNA simple Total RNA Kit (TianGen, Beijing) was used to extract RNA from tissues and PBMCs. Briefly, RNALater was carefully removed, and 1 ml of TRIzol was added to each tube. After phase separation by adding 200 μl chloroform and centrifugation, the aqueous phase was removed to a new tube. Moreover, 0.5-fold volume of anhydrous ethanol was added, mixed well, and transferred to the adsorption column. The column was deproteinized and washed with buffer PD, followed by two more washes with buffer PW. The column was dried at 12,000 × g for 2 min at room temperature, and 30 μl of RNase-free water was added to elute the RNA. RNA quantity and integrity were detected by NanoDrop2000 and Agilent2100 BioAnalyzer, respectively.

The first strand of cDNA was synthesized using M-MuLV reverse transcriptase, random oligonucleotide, and cDNA fragment. After degrading the RNA strand with RNase H, the DNA polymerase I system was used to synthesize the second strand. The purified double-strand cDNA underwent cDNA terminal repair, poly-A tail addition, connector connection, length screening, and PCR amplification and purification and finally obtained the library. The obtained cDNA fragments were evaluated using the Agilent BioAnalyzer 2100 system, and sequencing was performed on the Illumina platform.

### Transcriptomics Data Analysis

Clean reads were obtained by removing low-quality reads and ligand from the original sequence. The Q20/Q30 values and GC content were calculated to ensure quality. Clean reads were accurately aligned with the human reference genome (hg19) using HISAT2 software (Mortazavi et al., 2008), and the FeatureCounts in SubRead software (Liao et al., 2014) was used to calculate the read number. Read numbers lower than 10, unpaired reads, and reads in multiple regions of the genome were filtered out. Sequencing depth and gene length were corrected (Bray et al., 2016), and the expression values (FPKM) of all genes in each sample were calculated. To determine the associations between samples, we calculated the square of the Pearson correlation coefficient (*R* ^2^) of the samples within groups and performed principal component analysis (PCA) using the calculation square of linear algebra based on FPKM.

Expressed genes were defined as significant with the DESeq2 cutoff of *p* value < 0.05 and |log 2fold change |> 1. Meanwhile, clustering analysis and visualization were performed for differentially expressed genes (DEGs) using the R package “vocano.” Gene Ontology (GO) annotation and Kyoto Encyclopedia of Genes and Genomes (KEGG) pathway enrichment analysis were performed using the R package “clusterprofile” and web-based gene set analysis toolkit WebGestalt (Liao et al., 2019) based on DEGs.

To better interpret the expression data, the weighted gene co-expression network analysis (WGCNA) algorithm in integrated Differential Expression and Pathway analysis (Ge et al., 2018) was applied to perform the cluster analysis of samples at the same time. WGCNA constructed a clustering tree based on the correlation of gene expression and then divided it into modules. The target genes and gene networks could be identified by exploring the correlation between modules and phenotypes. The most variable genes to include were set as 1,000, and the minimum module size was set to 20. In addition, the corresponding module information was extracted to perform GO enrichment analysis and visualized with a bubble chart.

### Quantitative Polymerase Chain Reaction

7 pairs of corneal epithelial samples from patients and control donors were used for validation in RT-PCR. SYBR Green qPCR response system was 20 µl, including 2 × PerfectStart Green qPCR SuperMix (10 µl), cDNA (1 µl), and forward and reverse primer (0.4 µl), filled with water to total 20 µl. Furthermore, 45 cycles were set as follows: denaturation at 95°C for 5 s, annealing at 60°C for 30 s, and extension at 72°C for 10 s. The qPCR primer sequences for the detected genes are listed in Supplementary Table S10. 3 technical replicates were performed for each biological reaction and Graphpad Prism 6.0 was used for statistical analysis.

### Comparison to Keratoconus Candidate Genes in the Literature

Genomic studies of KTCN by searching PubMed and GWAS repositories were included if they identified candidate gene loci through linkage study, genome-wide association study, and whole-exome sequencing study in patients with KTCN. Candidate genes associated with KTCN published by the original author were collected, and this process identified 46 candidate genes from 36 articles combined with the database. Proteomics studies of KTCN were included if they identified protein expression changes in patients with KTCN. An extensive search of PubMed collected 12 proteomics studies that met the requirements (Supplementary Table S9). Using the original author’s statistical criteria, 1844 differentially expressed proteins were identified. We then compared our transcriptome data with candidate gene loci and a proteomics gene list and visualized them using the GO plot package.

### Identification of Differentially Splicing Events

Alternative splicing (AS) events were detected using two parallel approaches, rMATS (Shen et al., 2014) and CASH (Wu et al., 2018). The rMATS method quantifies the expression of AS events using a hierarchical model to simultaneously account for sampling uncertainty and variability. It calculates the *p*-value using a likelihood-ratio test to represent the difference in the inclusion level between groups of samples. The events with false discovery rate (FDR) < 0.05 were considered as significantly different between samples. Five types of splicing events were examined, including skipped exon (SE), alternative 3′ splice site (A3SS), alternative 5′ splice site (A5SS), mutually exclusive exons (MXE), and retained intron (RI). The CASH method directly self-constructs AS sites using the SpliceCons module from RNA-Seq data to explore novel AS events. Accordingly, SpliceDiff module merges the expression of AS inclusion/exclusion events and the expression of AS exons to infer differential levels of AS events between samples. The significant splice events (FDR < 0.05) detected in the patients were classified as cassette exon (i.e., skipped exon), multi-cassette exons, A3SS, A5SS, alternative start exon (AltStart), and alternative end exon (AltEnd), MXE and intron retention (IR i.e., retained intron). Differentially spliced genes were detected from differentially expressed alternative splicing events and were further applied for GO annotation.

### RNAseq Meta-Analysis

Two datasets (GSE77938, GSE112155) from the Gene Expression Omnibus (GEO) database were included in the meta-analysis. GSE77938 comprises 132 RNA-Seq profiles (25 cases with a later time-point RNA-seq data file being removed due to conversion failure, 25 controls); GSE112155 contains 20 RNA-Seq profiles (10 cases, 10 controls). RNA-seq data of the three databases were included to meta-analysis. Hisat2 (Kim et al., 2015) was used to assign RNA-seq data and FeatureCounts (Liao Y et al., 2014) was utilized to assemble KCTN RNA-seq data with control RNA-seq data to gain raw counts. Raw counts were then processed by Bioconductor packages in R v4.0.5 (www.r-project.org). We removed batch effect and other unwanted variation for RNA sequencing data by sva package. Normalization, DEGs analysis and annotation were conducted by preprocessCore package, DESeq2 package and org.Hs.eg.db respectively. Benjamini and Hochberg method control the False Discovery Rate (FDR) for multiple-testing adjustment. DEGs were defined as FDR < 0.05, and |log (fold-change) | >2. Gene Ontology (GO) annotation and Kyoto Encyclopedia of Genes and Genomes (KEGG) pathway enrichment analysis were conducted by R package “clusterprofile” and Webgestalt based on DEGs.

### Protein-Protein Interaction Analysis and Protein-Drug Prediction for Hub Genes

DEGs were imported into Network Analyst (Zhou et al., 2019) (https://www.networkanalyst.ca/) to construct a PPI network, PPI data were derived from STRING interactome (Szklarczyk et al., 2019) with a high confidence score of ≥ 900 and requirement for experimental evidence. Besides, the PPI network was visualized by Cytoscape (Cline et al., 2007), and hub genes were predicted via CytoHubba as previously described (Sun et al., 2021). Protein-chemical interaction analysis was performed based on data from the Comparative Toxicogenomics Database (CTD) (Mattingly et al., 2006) (downloaded on November 2016), a database identifies interactions between chemicals and genes, and promotes understanding about the effects of environmental chemicals on human health. Drugs and chemicals were ranked according to degree and betweenness.

## Results

### Assessments of Study Patients

We recruited patients with KTCN of East Asian ancestry from Tianjin, China (aged 12–19 years old). As controls for the KTCN samples, we used samples of mild myopia patients (aged 18–24 years old) from the same geographical and ethnic populations as cases. The mean age of patients with KTCN (16.00 ± 2.27 years) was comparable to that of the normal patients (20.14 ± 1.81 years). The corneal topography images showed significant differences in parameters between the two studied groups in anterior and posterior keratometry at flat axis (K1), steep axis (K2), and thinnest corneal thickness (Kmean) (Supplementary Table S1, Supplementary Figure S1).

The average RNA concentration and RNA integrity number value among tissue and blood samples were 265 ng/μl and 6.75 and 360.5 ng/μl and 6.47, respectively. Clean reads were obtained after raw data filtering, sequencing error rate assessment, and GC content distribution evaluation. The mean values of clean reads Q20 and Q30 were 97.96 and 94.26%, respectively. The sequencing error rates of the single bases were all less than 3% (Supplementary Table S2).

### Gene Expression Distribution and the Correlation Between Samples

**FIGURE 1:**
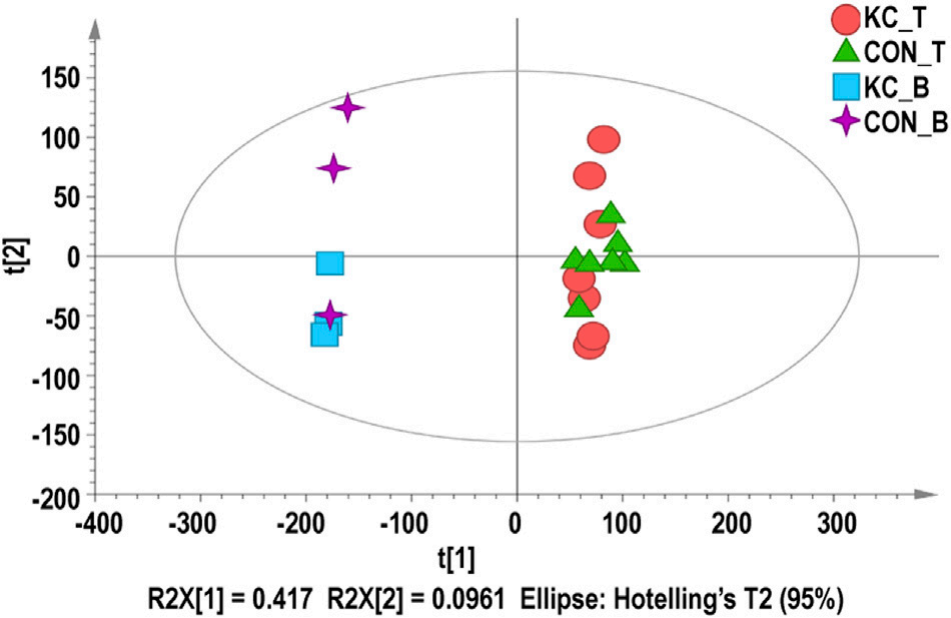
Principal component analysis (PCA) for corneal epithelial samples and blood samples. Red dots represent KTCN corneal epithelial samples, green triangles represent control corneal epithelial samples, blue squares represent KTCN blood samples, and purple crosses represent control blood samples.

After calculating the expression values FPKM of all the genes in each sample, the distribution of gene expression levels in different samples is shown in the box diagram (Supplementary Figures S2A, S2B). Across all samples, 12,187–12,665 unique protein-coding genes per sample with FPKM ≥ 1 were detected, and more than 351 genes were highly expressed (FPKM ≥ 60). The correlation analysis showed a high degree of Pearson correlation coefficient (*R* ^2^ = 0.795–0.969) between samples from patients with KTCN and control patients (Supplementary Figures S2C, S2D). PCA showed close clustering by tissue type (Figure 1).

### Dysregulated Pathways Between Keratoconus and Control Corneal Epithelium

**FIGURE 2:**
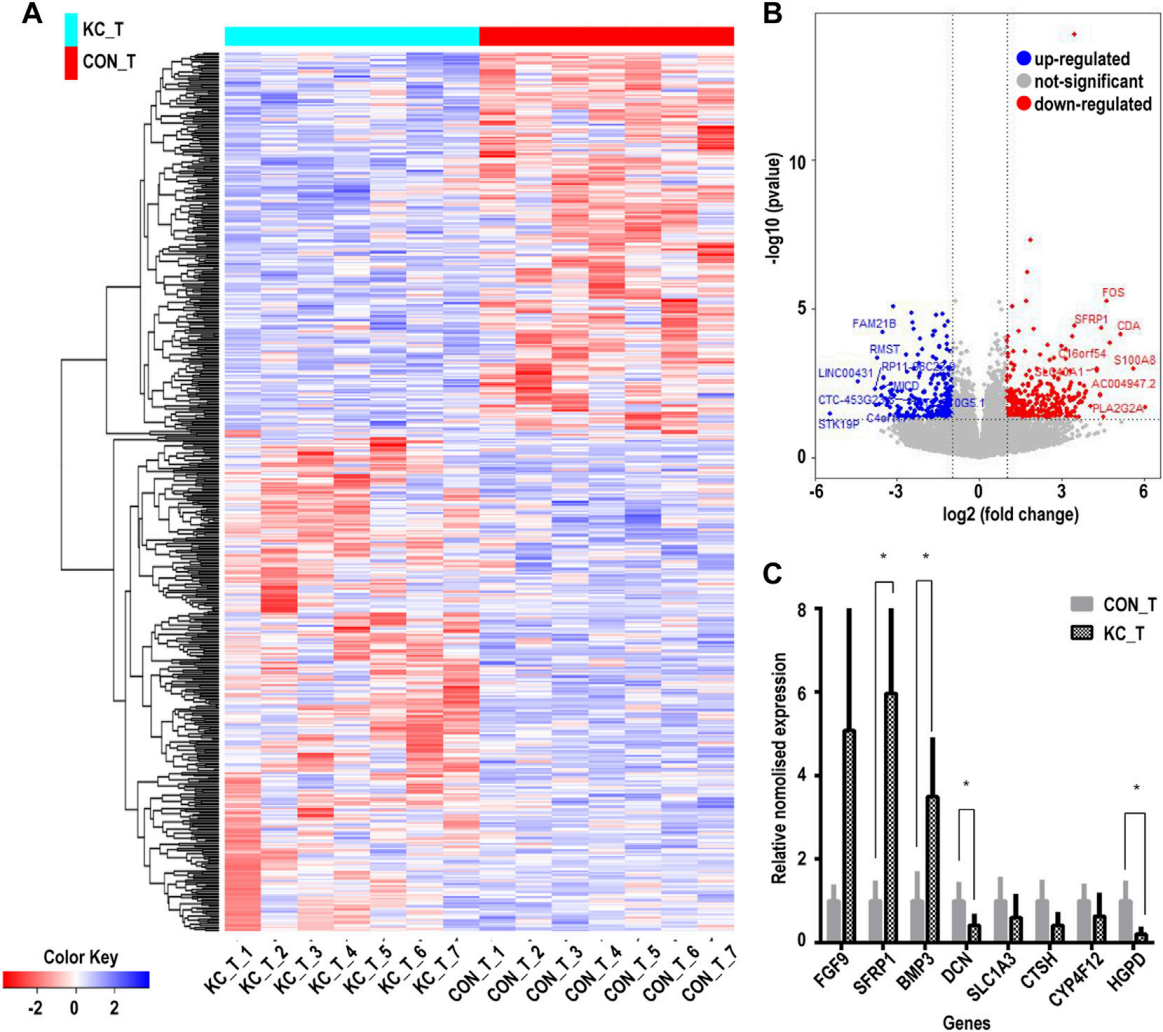
Cluster analysis of differentially expressed genes in corneal epithelial samples and RNA level expression validation. **(A)** Hierarchical clustering analysis of differentially expressed genes by a heatmap plot. Higher enrichment is colored in red, the lower ones are colored in blue. **(B)** Differentially expressed genes are shown by a volcano plot in corneal epithelial samples. Red dots indicate upregulated genes, and blue ones represent downregulated genes. **(C)** Eight genes were validated by qPCR. CON_T, control corneal epithelial samples, KC_T, KTCN corneal epithelial samples. (*p < 0.01).

Based on the cutoff mentioned in the method, 547 genes were identified as DEGs (308 upregulated and 239 downregulated) between KTCN and control corneal epithelial samples (Figures 2A,B; Supplementary Table S3). The expression levels of secreted frizzled-related protein 1 (SFRP1), TPPP3, KCNB2, RASSSF2, CD97, BMP3, SLC16A14, HLA-DPA1, LIX1, FGF12, GRIK5, CRIP2, FGF9, PRR11, and CDCA2 ranked top 15 significantly upregulated genes in the KTCN samples, whereas LAMP3, SEPP1, CTSH, CYP4F12, TGM5, PILRB, KCNJ2, HPGD, ADAM23, ZNF469, SLC1A3, FMO1, PTN, and FAM21B were the top 15 downregulated genes (Table 1).

**TABLE 1:** The top dysregulated differentially expressed genes in corneal epithelial samples of patients with keratoconus.

Differentially expressed	Gene symbol	Ensenbl ID	Description	Log2 (fold change)	*p* value	FDR
Down-regulated	LAMP3	ENSG00000078081	lysosomal associated membrane protein 3	−1.18	8.74E-05	5.71E-02
	SEPP1	ENSG00000250722	selenoprotein P	−1.20	8.54E-05	5.71E-02
	CTSH	ENSG00000103811	cathepsin H	−1.27	3.75E-05	4.49E-02
	CYP4F12	ENSG00000186204	cytochrome P450 family 4 subfamily F member 12	−1.32	1.50E-05	2.45E-02
	TGM5	ENSG00000104055	Transglutaminase 5	−1.44	2.10E-04	8.76E-02
	PILRB	ENSG00000121716	paired immunoglobin-like type 2 receptor beta	−1.46	1.82E-04	8.31E-02
	KCNJ2	ENSG00000123700	potassium voltage-gated channel subfamily J member 2	−1.62	5.82E-05	5.10E-02
	HPGD	ENSG00000164120	hydroxyprostaglandin dehydrogenase 15-(NAD)	−1.65	5.80E-05	5.10E-02
	ADAM23	ENSG00000114948	ADAM metallopeptidase domain 23	−1.69	7.79E-05	5.71E-02
	ZNF469	ENSG00000225614	zinc finger protein 469	−2.08	2.36E-04	8.76E-02
	SLC1A3	ENSG00000079215	solute carrier family 1 member 3	−2.17	1.04E-04	6.33E-02
	AXIN2	ENSG00000168646	axin 2	−2.40	4.70E-05	4.91E-02
	FMO1	ENSG00000010932	flavin containing monooxygenase 1	−2.46	1.38E-05	2.45E-02
	PTN	ENSG00000105894	Pleiotrophin	−3.15	8.62E-06	1.96E-02
	FAM21B	ENSG00000152726	WASH complex subunit 2A	−3.53	6.28E-05	5.24E-02
Up-regulated	SFRP1	ENSG00000104332	secreted frizzled related protein 1	4.46	4.31E-05	4.80E-02
	TPPP3	ENSG00000159713	tubulin polymerization promoting protein family member 3	3.47	5.77E-15	1.16E-10
	KCNB2	ENSG00000182674	potassium voltage-gated channel subfamily B member 2	3.47	3.81E-05	4.49E-02
	RASSF2	ENSG00000101265	Ras association domain family member 2	3.38	8.83E-05	5.71E-02
	CD97	ENSG00000123146	adhesion G protein-coupled receptor E5	2.50	1.67E-04	8.16E-02
	BMP3	ENSG00000152785	bone morphogenetic protein 3	1.99	4.90E-05	4.91E-02
	SLC16A14	ENSG00000163053	solute carrier family 16 member 14	1.88	4.75E-08	4.76E-04
	HLA-DPA1	ENSG00000231389	major histocompatibility complex, class II, DP alpha 1	1.72	5.43E-06	1.69E-02
	LIX1	ENSG00000145721	limb and CNS expressed 1	1.66	2.75E-04	9.35E-02
	FGF12	ENSG00000114279	fibroblast growth factor 12	1.24	2.86E-04	9.35E-02
	GRIK5	ENSG00000105737	glutamate ionotropic receptor kainate type subunit 5	1.22	8.79E-06	1.96E-02
	CRIP2	ENSG00000182809	cysteine rich protein 2	1.06	8.60E-05	5.71E-02
	FGF9	ENSG00000102678	fibroblast growth factor 9	1.04	3.32E-04	9.88E-02
	PRR11	ENSG00000068489	proline rich 11	1.02	1.19E-04	7.01E-02
	CDCA2	ENSG00000184661	cell division cycle associated 2	1.01	2.10E-04	8.76E-02

The DEGs were validated by randomly selecting eight genes from the top 30 identified DEGs. Among the tested DEGs, consistent with our RNA-Seq findings, SFRP1, FGF9, and BMP3were significantly upregulated in the KTCN epithelium. In contrast, SLC1A3, HPDG, DCN, CTSH, and CYP4F1 RNA expressions were downregulated in the KTCN epithelium (Figure 2C).

**FIGURE 3:**
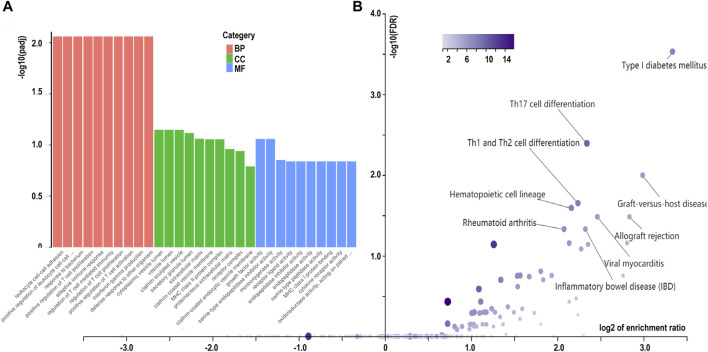
Enrichment analysis of differentially expressed genes in corneal epithelial samples. **(A)** Gene Ontology analysis of differentially expressed genes. Biological process (BP) is colored in red. Cellular component (CC)is colored in green. Molecular function (MF) is colored in blue. **(B)** Kyoto Encyclopedia of Genes and Genomes pathway enrichment analysis based on differentially expressed genes. CON, control samples, KC, KTCN samples.

The GO annotation of the 547 DEGs revealed that the biological processes of many DEGs were related to leukocyte cell-cell adhesion (such as HLA-DPA1 and S100A8), regulation of lymphocyte activation (such as SFRP1and CD247), and adaptive immune responses (such as CTSH and LAMP3) (Figure 3A; Table 2; Supplementary Table S4). Meanwhile, immune- and inflammatory-related KEGG pathways, including Th17 cell differentiation, Th1 and Th2 cell differentiation, and IL-17 signaling pathway, were observed in the DEGs of the samples (Figure 3B; Table 3; Supplementary Table S5). The enrichment of inflammatory immune diseases, such as rheumatoid arthritis, bowel disease, and autoimmune thyroid disease, was also observed, some of which have been reported to be a comorbidity accompanied by KTCN previously (Nemet et al., 2010; Tréchot et al., 2015).

**TABLE 2:** The significantly biological process of Gene Ontology analysis for 547 differentially expressed genes.

Differentially expressed	Gene symbol	Ensenbl ID	Description	Log2 (fold change)	P value	FDR
Down-regulated	LAMP3	ENSG00000078081	lysosomal associated membrane protein 3	−1.18	8.74E-05	5.71E-02
	SEPP1	ENSG00000250722	selenoprotein P	−1.20	8.54E-05	5.71E-02
	CTSH	ENSG00000103811	cathepsin H	−1.27	3.75E-05	4.49E-02
	CYP4F12	ENSG00000186204	cytochrome P450 family 4 subfamily F member 12	−1.32	1.50E-05	2.45E-02
	TGM5	ENSG00000104055	Transglutaminase 5	−1.44	2.10E-04	8.76E-02
	PILRB	ENSG00000121716	paired immunoglobin-like type 2 receptor beta	−1.46	1.82E-04	8.31E-02
	KCNJ2	ENSG00000123700	potassium voltage-gated channel subfamily J member 2	−1.62	5.82E-05	5.10E-02
	HPGD	ENSG00000164120	hydroxyprostaglandin dehydrogenase 15-(NAD)	−1.65	5.80E-05	5.10E-02
	ADAM23	ENSG00000114948	ADAM metallopeptidase domain 23	−1.69	7.79E-05	5.71E-02
	ZNF469	ENSG00000225614	zinc finger protein 469	−2.08	2.36E-04	8.76E-02
	SLC1A3	ENSG00000079215	solute carrier family 1 member 3	−2.17	1.04E-04	6.33E-02
	AXIN2	ENSG00000168646	axin 2	−2.40	4.70E-05	4.91E-02
	FMO1	ENSG00000010932	flavin containing monooxygenase 1	−2.46	1.38E-05	2.45E-02
	PTN	ENSG00000105894	Pleiotrophin	−3.15	8.62E-06	1.96E-02
	FAM21B	ENSG00000152726	WASH complex subunit 2A	−3.53	6.28E-05	5.24E-02
Up-regulated	SFRP1	ENSG00000104332	secreted frizzled related protein 1	4.46	4.31E-05	4.80E-02
	TPPP3	ENSG00000159713	tubulin polymerization promoting protein family member 3	3.47	5.77E-15	1.16E-10
	KCNB2	ENSG00000182674	potassium voltage-gated channel subfamily B member 2	3.47	3.81E-05	4.49E-02
	RASSF2	ENSG00000101265	Ras association domain family member 2	3.38	8.83E-05	5.71E-02
	CD97	ENSG00000123146	adhesion G protein-coupled receptor E5	2.50	1.67E-04	8.16E-02
	BMP3	ENSG00000152785	bone morphogenetic protein 3	1.99	4.90E-05	4.91E-02
	SLC16A14	ENSG00000163053	solute carrier family 16 member 14	1.88	4.75E-08	4.76E-04
	HLA-DPA1	ENSG00000231389	major histocompatibility complex, class II, DP alpha 1	1.72	5.43E-06	1.69E-02
	LIX1	ENSG00000145721	limb and CNS expressed 1	1.66	2.75E-04	9.35E-02
	FGF12	ENSG00000114279	fibroblast growth factor 12	1.24	2.86E-04	9.35E-02
	GRIK5	ENSG00000105737	glutamate ionotropic receptor kainate type subunit 5	1.22	8.79E-06	1.96E-02
	CRIP2	ENSG00000182809	cysteine rich protein 2	1.06	8.60E-05	5.71E-02
	FGF9	ENSG00000102678	fibroblast growth factor 9	1.04	3.32E-04	9.88E-02
	PRR11	ENSG00000068489	proline rich 11	1.02	1.19E-04	7.01E-02
	CDCA2	ENSG00000184661	cell division cycle associated 2	1.01	2.10E-04	8.76E-02

**TABLE 3:** The significantly enriched Kyoto Encyclopedia of Genes and Genomes pathways for 547 differentially expressed genes.

KEGGID	Description	*p*-value	Padj	Count	Genes
hsa04940	Type I diabetes mellitus	3.88382E-05	0.00,815,602	5	IL1B/PRF1/CPE/GAD1/GZMB
hsa04659	Th17 cell differentiation	0.002095981	0.17,743,168	7	FOS/CD247/IL1B/JUN/IL23R/JAK3/IRF4
hsa04650	Natural killer cell mediated cytotoxicity	0.002,534,738	0.17,743,168	7	CD247/LCP2/PRF1/RAC2/GZMB/ITGAL/CD244
hsa05332	Graft-versus-host disease	0.003,783,333	0.19,862,497	3	IL1B/PRF1/GZMB
hsa04657	IL-17 signaling pathway	0.005,176,762	0.217424	6	FOS/S100A8/IL1B/JUN/S100A9/CXCL2
hsa04662	B cell receptor signaling pathway	0.011,318,793	0.25,962,967	5	FOS/JUN/RAC2/IFITM1/PIK3AP1
hsa04640	Hematopoietic cell lineage	0.016,882,535	0.25,962,967	5	IL1B/CD38/IL7R/MME/CSF1R
hsa04658	Th1 and Th2 cell differentiation	0.016,882,535	0.25,962,967	5	FOS/CD247/RUNX3/JUN/JAK3
hsa04060	Cytokine-cytokine receptor interaction	0.022,561,212	0.29,611,591	9	IL1B/IL10RA/IL7R/CX3CR1/IL23R/CCR4/CCR3/CXCL2/CSF1R
hsa05330	Allograft rejection	0.031,802,321	0.3,928,522	2	PRF1/GZMB
hsa04010	MAPK signaling pathway	0.042,545,508	0.39,738,129	10	FOS/FGF12/FGF9/CACNA2D1/IL1B/JUN/RAC2/IGF2/HSPA6/CSF1R
hsa04725	Cholinergic synapse	0.043,522,713	0.39,738,129	5	FOS/KCNJ2/SLC18A3/PIK3R5/CHAT
hsa05323	Rheumatoid arthritis	0.034,883,144	0.39,738,129	4	FOS/IL1B/JUN/ITGAL

**FIGURE 4:**
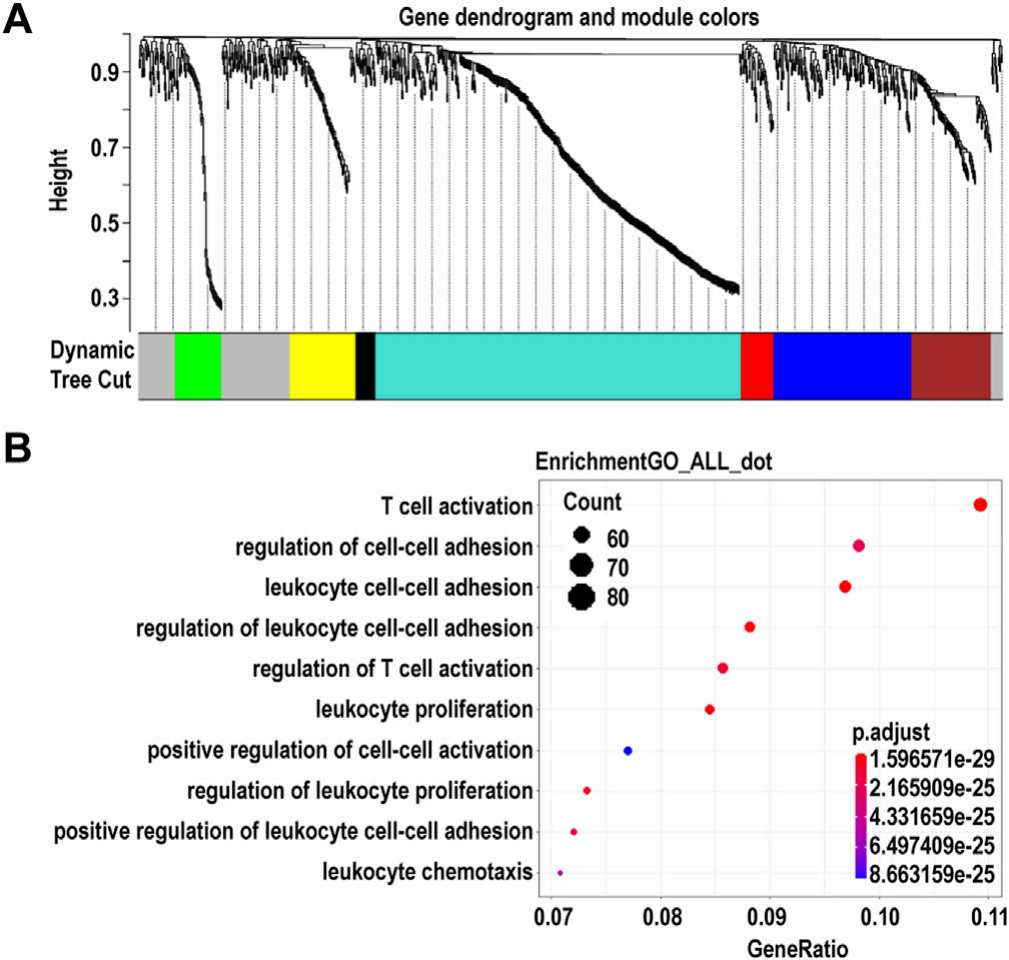
Identification of modules and functional annotation analysis for the module genes in corneal epithelial samples. **(A)** Module detection for the expressed genes of corneal epithelial samples. **(B)** Gene Ontology analysis for the turquoise module genes.

Using WGCNA, we identified seven co-expression modules that were constructed from the 896 genes. These co-expression modules were constructed and are shown in different colors (Figure 4A). The number of genes in these modules ranged from 23 to 424 (Supplementary Table S6). We found that the largest turquoise module was significantly correlated with the immune system processes. GO enrichment analysis was performed on the genes in the largest turquoise module. Genes in the turquoise module were mainly enriched in GO:0002283 (neutrophil activation involved in immune response), GO:0043312 (neutrophil degranulation), and GO:0002429 (immune response-activating cell surface receptor signaling pathway) (Figure 4B).

### Expression Changes in the Blood of Patients With Keratoconus Emphasize Immune Regulation

**FIGURE 5:**
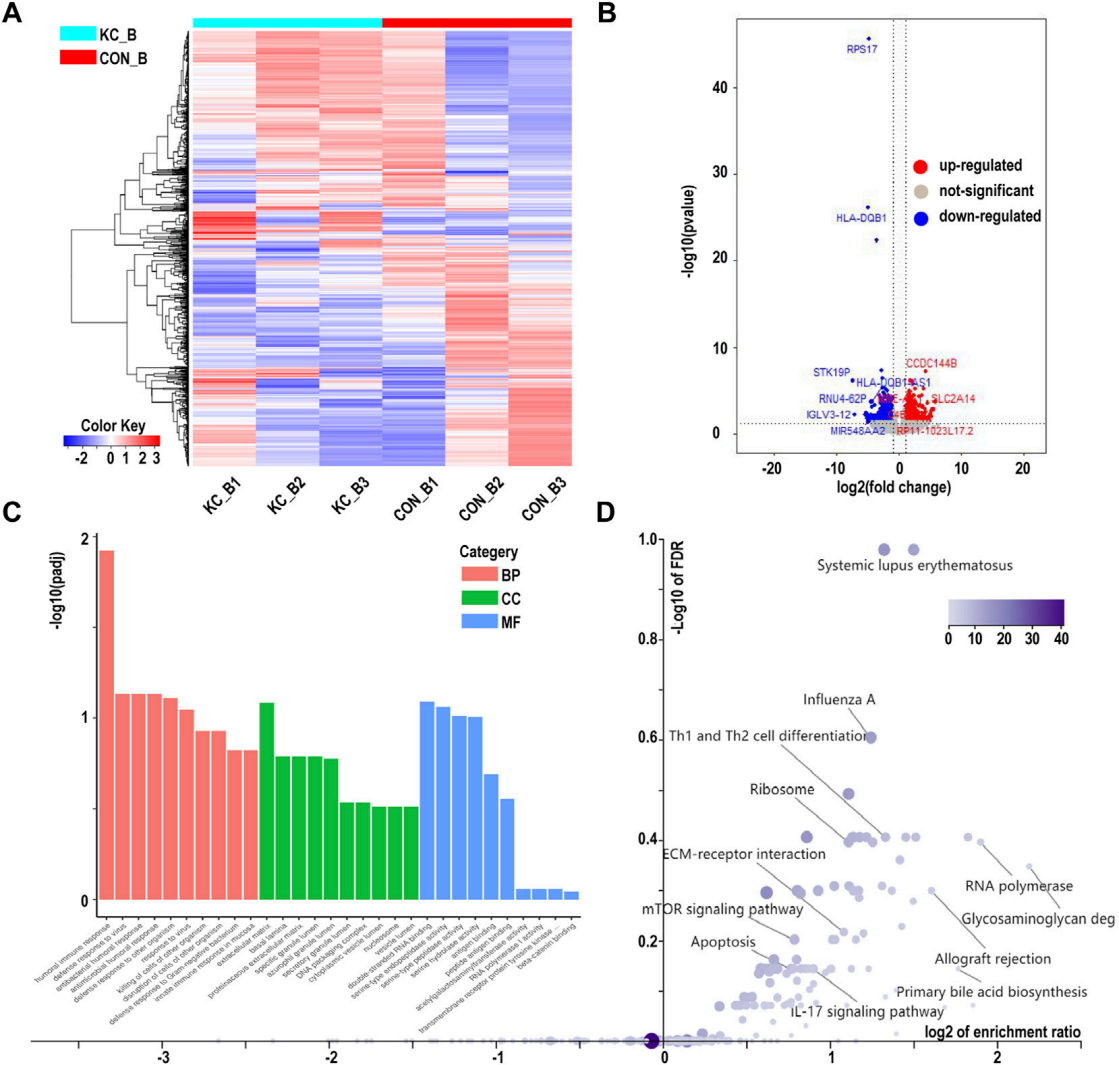
Hierarchical cluster analysis of differentially expressed genes in blood samples. **(A)** Hierarchical clustering analysis of differentially expressed genes by a heatmap plot. Higher enrichment is colored in red, the lower ones are colored in blue. **(B)** Differentially expressed genes were showed by a volcano plot in blood samples. Red dots represent upregulated genes, and blue ones indicate downregulated genes. **(C)** Gene Ontology analysis of differentially expressed genes. **(D)** Kyoto Encyclopedia of Genes and Genomes pathway enrichment analysis based on differentially expressed genes.

We next determined whether networks of KTCN whole blood have biological processes similar to those of the corneal epithelium. RNA-Seq and enrichment annotations were performed for peripheral blood using the same method as that used for the tissue. Of the 28,080 genes analyzed, 509 genes were upregulated and 622 were downregulated in blood samples from patients with KTCN versus controls (Figures 5A,B). We found a significant excess of biological processes to be significantly enriched in the humoral immune response (padj < 0.05). The changes were prominent in genes related to HLA-DQB1, LTF, and PAX5. (Figures 5C,D; Supplementary Tables S3–S5).

We then explored molecules that act in the same direction in the whole blood and corneal epithelium. The overlap in dysregulated genes was not significantly larger than expected. However, it still has important implications, allowing us to determine common key factors between the two systems. Genes, such as TUBB1 and BANK1, that significantly (*p* < 0.05) enriched for dysregulation in both the corneal epithelium and whole blood are listed in Supplementary Table S7.

### Co-Analysis of Previous Genomics and Proteomics Study of Keratoconus Reinforced the Conjecture

**FIGURE 6:**
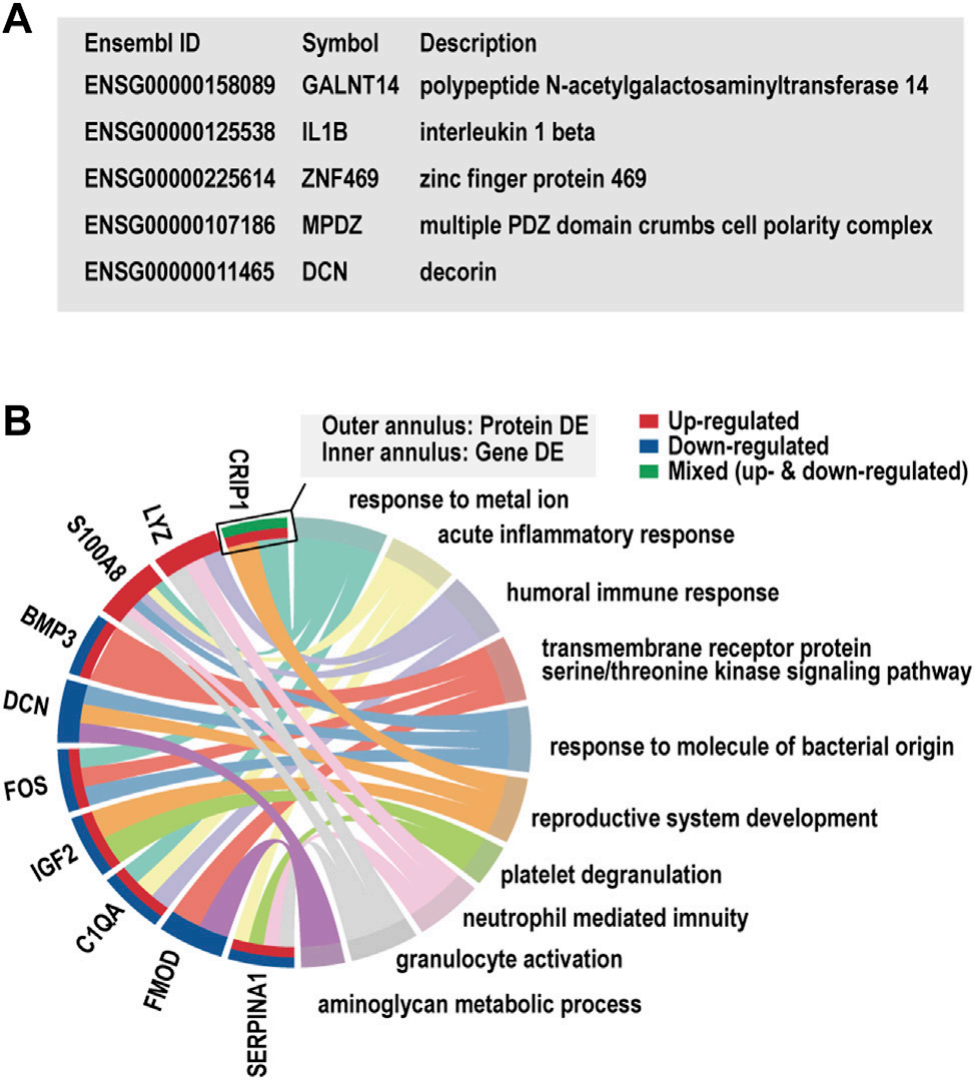
Overlap gene and Gene Ontology (GO) cluster analysis. **(A)** The list of candidate gene loci that were also differentially expressed in our transcriptome study. **(B)** The enriched GO clusters for the dysregulated genes in our transcriptome and previous proteomics studies.

5 and 10 DEGs overlapped with the genetic candidate loci and the proteomics gene list, respectively (Figures 6A,B). We noted the inflammatory factor IL1B in the limited overlapping genes between genetic candidate loci and proteomics gene list. In addition, the GO clusters enriched in the list of overlapping genes from proteomics studies and our transcriptome study displayed evident immune and inflammatory involvement, which included acute inflammatory response (S100A8, C1QA, SERPINA1), humoral immune response (LYZ, S100A8, C1QA), and neutrophil-mediated immunity (LYZ, S100A8, SERPINA1).

### Alternative Splicing Events in Patients With Keratoconus

**FIGURE 7:**
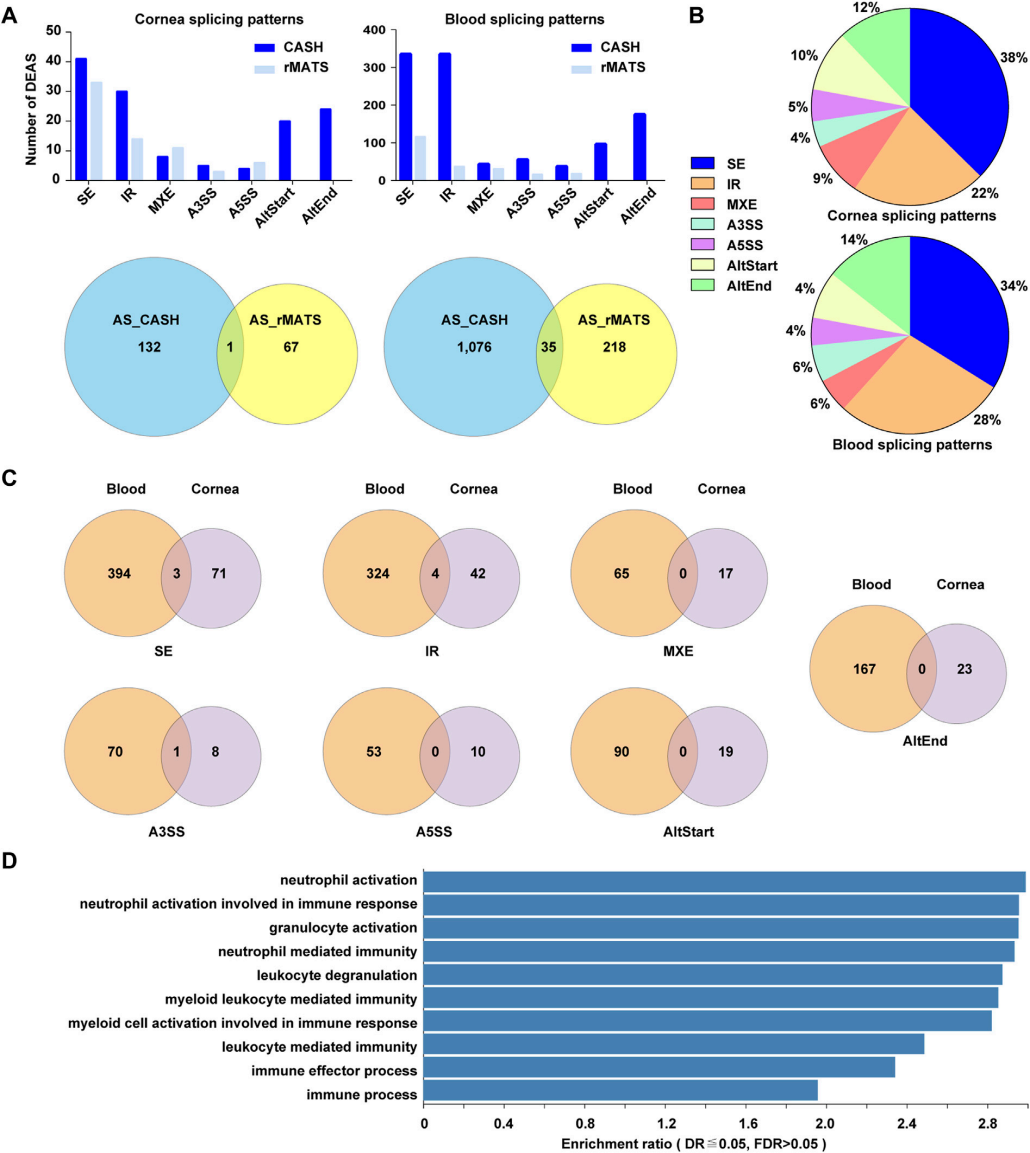
An overview of alternative splicing events in corneal epithelial and blood samples. **(A)**. The significantly differentially spliced events detected by two methods. **(B)** Pie charts showing the types and relative proportion of alternative splicing events in corneal epithelial and blood samples. **(C)** Venn diagrams depicting overlaps and differences between the events in corneal and blood samples. **(D)** The top 10 enriched GO biological processes among differentially spliced genes in blood samples.

We also sought to identify transcripts that were differentially spliced between KTCN and control patients 67 candidate aberrant splicing events were identified with a threshold of FDR <0.05 by rMATS in cornea samples with KCTN. CASH detects more aberrant splicing events up to 132 compared to rMATS. On the other hand, CASH and rMATS method showed 1,076 and 218 differential splicing events in blood samples, among which 35 events were identified by both methods (Figure 7A).

The current study revealed 1,163 and 190 alternative splicing events in blood and cornea. The alternative splicing events in corneal samples included SE (38％), IR (22％), AltEnd (12％), AltStart (10％), MXE (9％), A5SS (5％) and A3SS (4％). In blood sample, the corresponding sequence was: SE (34%) > IR > AltEnd> AltStart > MXE> A3SS > A5SS (Figure 7B). 3 /71 SE, 4 / 42 IR, and 1 / 8 A3SS events overlapped in corneal and blood samples. The changes of A5SS, AltStart, AltEnd, and MXE were totally different between corneal epithelium and blood (Figure 7C).

The overrepresentation analysis for affected splicing events in corneal epithelial samples revealed several processes, but with high *p*-value. A few processes for aberrant splicing events in blood samples were captured in our functional annotation, including neutrophil activation, neutrophil activation involved in immune response, granulocyte activation, and neutrophil mediated immunity (Figure 7D).

### Mata-Analysis of GEO RNA-Seq Data of Keratoconus Confirms Our Suspicion

Through meta-analysis and enrichment analysis performed on the two previously reported datasets, again, Th17 cell differentiation, T-cell receptor signaling pathway, and IL-17 signaling were found to be enriched in DEGs of KCTN. This confirmed that the change of immune gene expression and the disorder of immune-related signaling pathways may mediate the molecular mechanism of KCTN. We also observed the enrichment of the “ECM receptor interaction” pathway, which is consistent with previous reports that extracellular matrix degradation may be involved in the pathogenesis of KCTN (Supplementary Tables S3–S5; Supplementary Figure S3).

### Potential Therapeutic Targets for Keratoconus

**FIGURE 8:**
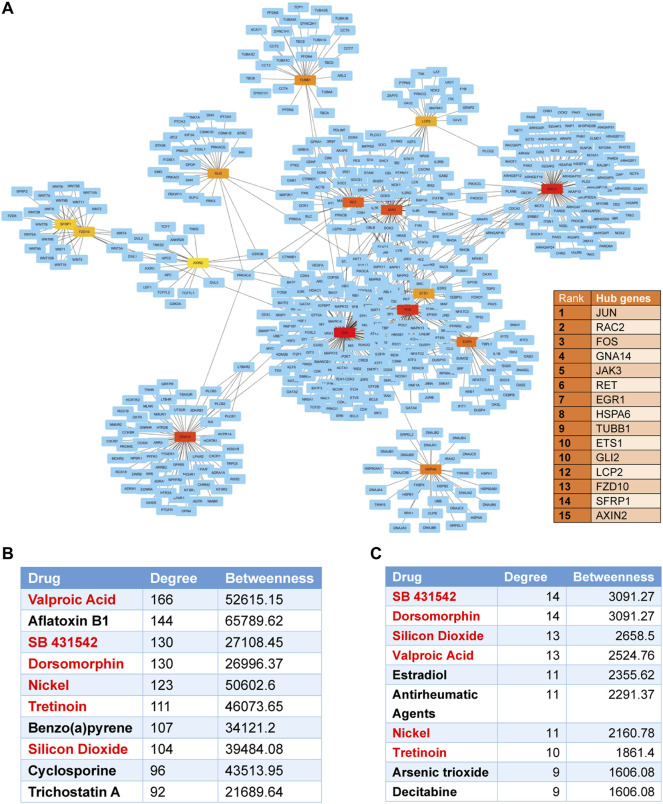
Protein-protein and protein-chemical interaction analysis for differentially expressed genes. **(A)** The protein-protein interaction (PPI) network and the top 15 hub genes in the network ranked by the Degree method. Nodes indicate the number of predicted gene interactions. Red squares are the hub genes. **(B)** The top 10 drugs associated with KTCN DEGs in the protein-chemical interactions network. **(C)** The top 10 drugs associated with hub genes. Overlapped drugs in two figures were marked in red.

On analyzing the DEGs using the GDA interface of NetworkAnalyst to acquire subnetworks (at least 3 nodes), there were 29 subnetworks created of which only 1 (subnetwork 1) had a seed number above 3. The subnetwork1 was identified with 806 nodes and 1,092 connection edges. We used Cytoscape software to visualize the subnetwork1 (Figure 8A). The top 15 DEGs (JUN, RAC2, FOS, GNA14, JAK3, RET, EGR1, HSPA6, TUBB1, ETS1, GLI2, LCP2, FZD10, SFRP1, and AXIN2) were regarded as hub genes by node degrees. Among them, JUN was the most highly ranked gene (degree = 129; betweenness = 80856.21).

The protein–chemical interaction analysis of DEGs help to explore potential therapeutic drugs in treating KTCN. A single network was identified with 1887 nodes and 8,116 edges. 1,470 chemicals were identified to interact with DEGs. The top ten compounds associated with KTCN DEGs were predicted in Figure 8B. We also found that valproic acid, SB431542, and dorsomorphin were drugs that could interact with most hub genes. The top ten compounds which interact with hub genes were listed in Figure 8C.

## Discussion

Initial support for immune evidence in KTCN was obtained in an earlier proteomics study of KTCN tear samples in which immunoglobulin kappa chain (Lema et al., 2010) and polymeric immunoglobulin receptor (Balasubramanian et al., 2013) were decreased. Additionally, Nielsen et al., detected an increase in major histocompatibility complex class II, DR alpha (HLA-DRA1), using an expression microarray (Nielsen et al., 2003). In a recent study of two KTCN patient groups, using the canonical pathways method, researchers detected a large percentage of the RA pathway in the Middle Eastern ancestry group, emphasizing a dysregulation in inflammation and immune signals in the cornea (Shinde et al., 2020).

In the current study, we found evidence of the involvement of immune-inflammatory substances in the corneal epithelial transcriptome. Except for HLA-DPA1 and PILRB, SFRP1 was upregulated. SFRP1, which is an antagonist of Wnt signaling, is dysregulated in many studies concerning KTCN (Sutton et al., 2010; You et al., 2013a; You et al., 2013b; Iqbal et al., 2013; Khaled et al., 2018). SFRP1 participates in cell proliferation, migration, and differentiation. It might also serve as an anti-inflammatory factor by modulating the balance between pro-inflammatory and anti-inflammatory cytokines (Barandon et al., 2011). SFRP1 was reported to function in Th17 cell differentiation and was significantly correlated with IL-17 levels in patients with rheumatoid arthritis (Lee et al., 2012).

The internal consistency of our data between the blood and corneal epithelial transcriptomes allowed us to verify immune responses as core pathogenic changes in KTCN. The changes were prominent in humoral immune genes, such as HLA-DQB1 and LTF. Taking the LTF as an example in our study, downregulation of LTF was identified in the blood of patients with KTCN (Lema et al., 2010; Balasubramanian et al., 2012b; Priyadarsini et al., 2014). LTF, an iron-binding protein, is considered to play an important role in modifying innate and adaptive immune responses (Moreno-Expósito et al., 2018). LTF can act on antigen-presenting cells (Puddu et al., 2009) to mature dendritic cells, activate macrophages, accelerate antigen processing, and increase NK cell cytotoxicity. By acting on the maturation process of T lymphocytes, LTF can induce CD4 expression and direct differentiation of immature T lymphocytes toward CD4 + T lymphocytes, thus regulating the functional ability of T lymphocytes (Siqueiros-Cendón et al., 2014). LTF can promote TH1 response and inhibit the TH2 response, thereby activating cellular response and reducing the release of inflammatory factors. In addition, various clinical trials have found that LTF may exert anti-inflammatory effects by controlling TNF-α (Drago-Serrano et al., 2017). Knockout of LTF mice (LTF-KO) has been shown to be deficient in hematopoietic and immune systems. Neutrophil maturation, migration, phagocytosis, granule release, and antimicrobial response to bacterial challenge are affected in LTF-KO mice (Ward et al., 2008).

Although many studies have examined the changes in KTCN corneas, few have analyzed the blood samples of these patients, especially by transcriptome analysis. Our study, for the first time, quantified the gene expression profile in the whole blood of patients with KTCN, which allowed us to reexamine the mechanism of KTCN from the perspective of system theory. In addition, dysregulated genes in the blood may yield early biomarkers for KTCN. Most importantly, we have now found evidence to propose an “immune-inflammatory hypothesis” in KTCN. Using multilevel transcriptome data, we hypothesize that KTCN is a systemic disease with immune component and inflammatory basis.

A few limitations of this study must be considered. First, our experiment was restricted by the number of samples, and more samples from patients and controls are needed to increase the statistical power. Second, the corneal epithelium was used rather than the whole cornea button because of the limitation of the surgical procedure. As the most prominent layer of lesion, the biological data for the stromal layer of keratoconus are undoubtedly important. Further studies to perform on the corneal stroma lever if the surgical materials allowed and validate the protein levels of the altered gene expression identified in our study should be conducted. Based on the current results, it is also necessary to perform the next targeted molecular function experiments and discover potential biomarkers for KTCN detection and treatment.

In summary, we performed a comprehensive analysis of the expression profile in the corneal epithelium and peripheral blood of patients with KTCN and control donors to understand the pathogenesis of KTCN. A total of 547 DEGs were identified in the tissue expression profiles. Functional enrichment analysis showed that these DEGs were mainly related to leukocyte cell-cell adhesion, regulation of lymphocyte activation, and adaptive immune response. Concordantly, DEGs were significantly enriched in Th17 cell differentiation, Th1 and Th2 cell differentiation, and IL-17 signaling pathways. Meanwhile, through transcriptomics analysis of peripheral blood samples and co-analysis of previously reported omics studies, enrichment of immune-related biological processes was also observed. For the first time, RNA-Seq was used to compare gene expression in both the corneal epithelial and blood samples. Our results indicated the potential involvement of immune pathways in KTCN pathogenesis.

## Data Availability

RNA seq data were deposited in the NCBI Sequence Read Archive (SRA) under accession number: PRJNA799648.
