# Supplementary figures and images for: Comprehensive Transcriptome Analysis of Patients With Keratoconus Highlights the Regulation of Immune Responses and Inflammatory Processes
Source: Front Genet. 2022 Feb 25;13:782709. doi: 10.3389/fgene.2022.782709 (PMC8914074; doi:10.3389/fgene.2022.782709)

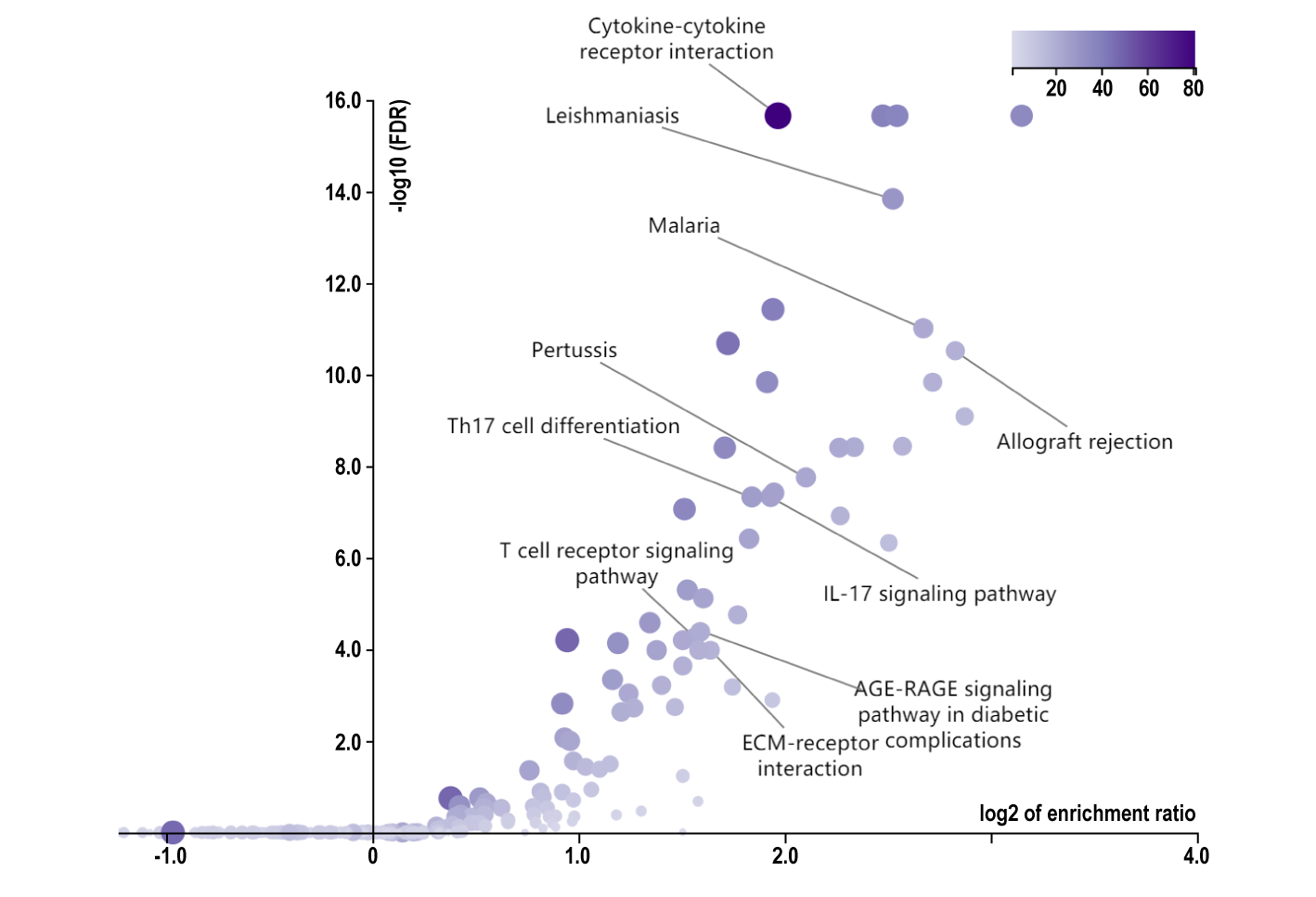

Supplement: Supplementary file 1 [file Image3.TIF]

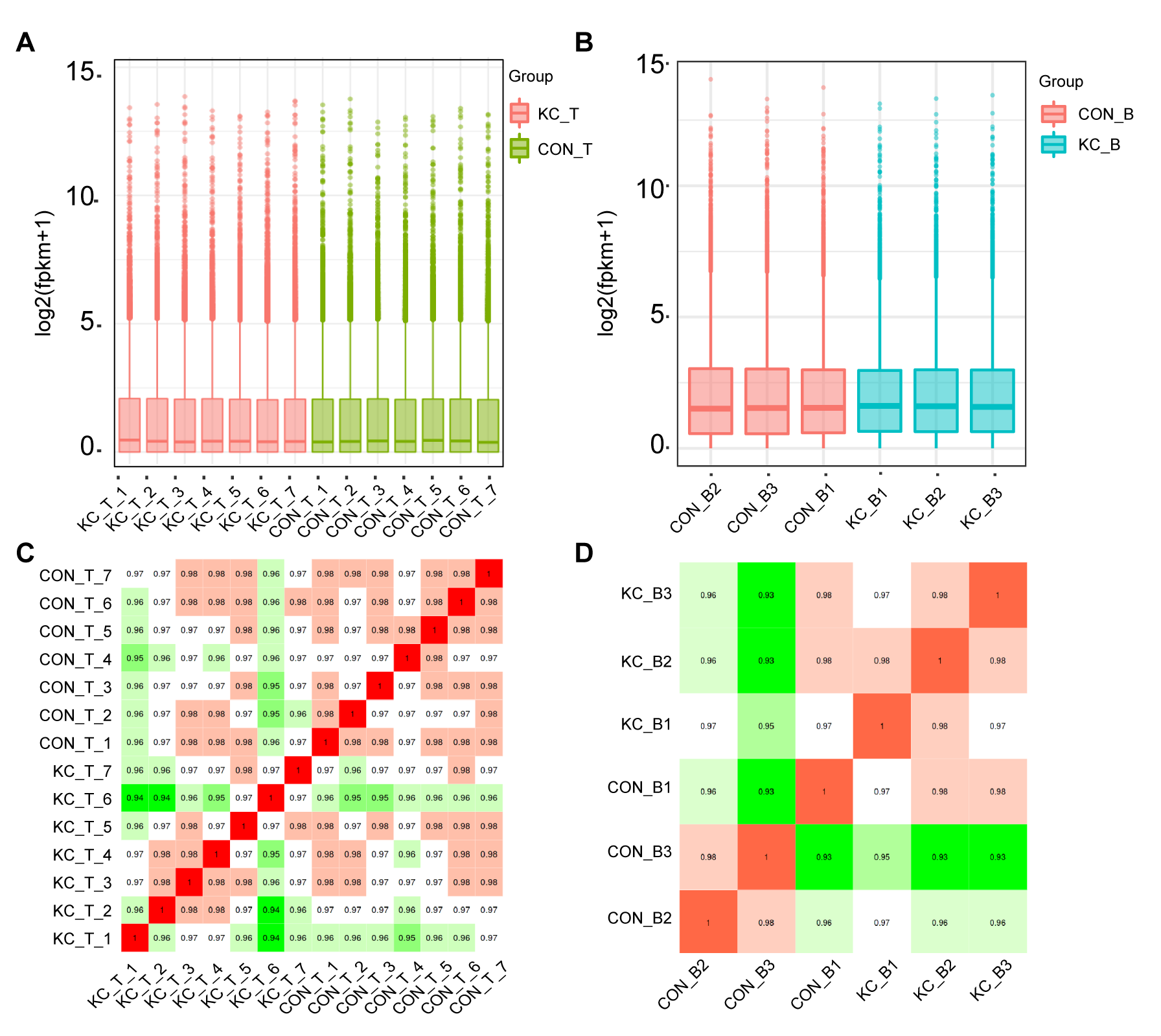

Supplement: Supplementary file 2 [file Image2.TIF]

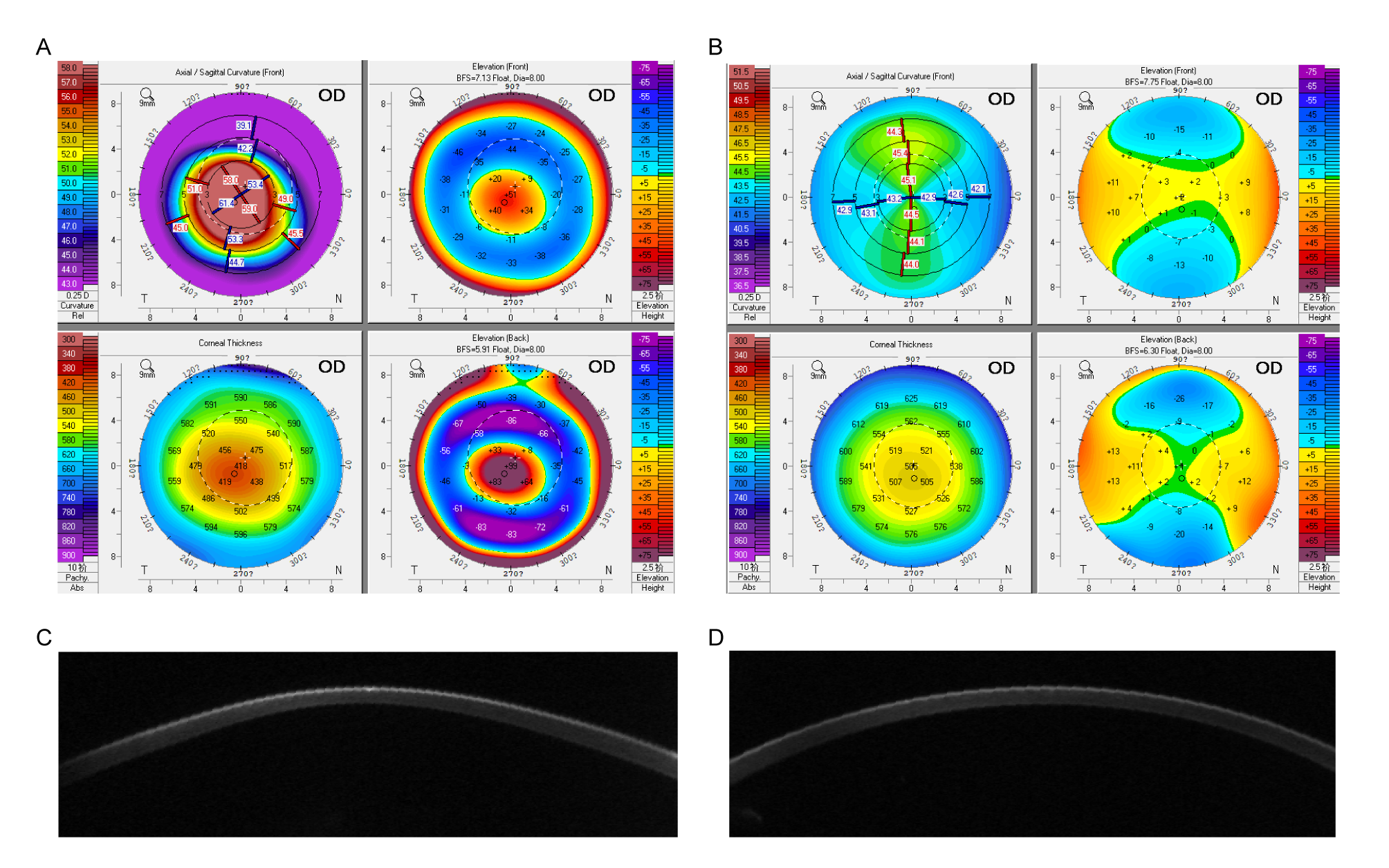

Supplement: Supplementary file 3 [file Image1.TIF]
